# Diffuse correlation spectroscopy measurements of blood flow using 1064 nm light

**DOI:** 10.1117/1.JBO.25.9.097003

**Published:** 2020-09-29

**Authors:** Stefan A. Carp, Davide Tamborini, Dibbyan Mazumder, Kuan-Cheng (Tony) Wu, Mitchell R. Robinson, Kimberly A. Stephens, Oleg Shatrovoy, Niyom Lue, Nisan Ozana, Megan H. Blackwell, Maria A. Franceschini

**Affiliations:** a Massachusetts General Hospital, Harvard Medical School, Optics at Athinoula A. Martinos Center for Biomedical Imaging, Department of Radiology, Charlestown, Massachusetts, United States; b Boston University, Department of Biomedical Engineering, Boston, Massachusetts, United States; c MIT, Health Sciences and Technology Program, Cambridge, Massachusetts, United States; d MIT Lincoln Laboratory, Lexington, Massachusetts, United States

**Keywords:** near-infrared, short-wave infrared, diffuse correlation spectroscopy, blood flow, Monte Carlo simulations

## Abstract

**Significance:** Diffuse correlation spectroscopy (DCS) is an established optical modality that enables noninvasive measurements of blood flow in deep tissue by quantifying the temporal light intensity fluctuations generated by dynamic scattering of moving red blood cells. Compared with near-infrared spectroscopy, DCS is hampered by a limited signal-to-noise ratio (SNR) due to the need to use small detection apertures to preserve speckle contrast. However, DCS is a dynamic light scattering technique and does not rely on hemoglobin contrast; thus, there are significant SNR advantages to using longer wavelengths (>1000  nm) for the DCS measurement due to a variety of biophysical and regulatory factors.

**Aim:** We offer a quantitative assessment of the benefits and challenges of operating DCS at 1064 nm versus the typical 765 to 850 nm wavelength through simulations and experimental demonstrations.

**Approach:** We evaluate the photon budget, depth sensitivity, and SNR for detecting blood flow changes using numerical simulations. We discuss continuous wave (CW) and time-domain (TD) DCS hardware considerations for 1064 nm operation. We report proof-of-concept measurements in tissue-like phantoms and healthy adult volunteers.

**Results:** DCS at 1064 nm offers higher intrinsic sensitivity to deep tissue compared with DCS measurements at the typically used wavelength range (765 to 850 nm) due to increased photon counts and a slower autocorrelation decay. These advantages are explored using simulations and are demonstrated using phantom and *in vivo* measurements. We show the first high-speed (cardiac pulsation-resolved), high-SNR measurements at large source–detector separation (3 cm) for CW-DCS and late temporal gates (1 ns) for TD-DCS.

**Conclusions**: DCS at 1064 nm offers a leap forward in the ability to monitor deep tissue blood flow and could be especially useful in increasing the reliability of cerebral blood flow monitoring in adults.

## 1 Introduction

Diffuse optical methods have been used for more than 30 years to noninvasively quantify hemodynamics in the brain, skeletal muscles, and other tissues. In particular, near-infrared spectroscopy (NIRS) is widely adopted for cerebral oximetry clinical applications^1^ and functional neuroimaging studies.^2^ NIRS employs light in the red and near-infrared spectral region, between 650 and 900 nm, where overall tissue absorption is low and dominated by hemoglobin species while water absorption is not significant. This optical window permits light penetration of several centimeters into the tissue and high sensitivity to hemoglobin concentration changes. Diffuse correlation spectroscopy (DCS)^3^^,^^4^ is another diffuse optical method that is rapidly growing and being employed in a range of biomedical applications.^5^^–^^7^ In DCS, a tissue of interest is illuminated by coherent near-infrared light, which causes a speckled interference pattern to form after the light scatters multiple times through the tissue. Dynamic scattering of the light by moving red blood cells causes the speckle pattern to fluctuate rapidly. These fluctuations are typically detected 2 to 3 cm away from the source and are quantified by measuring the temporal intensity autocorrelation curve, $g_{2}(\tau )=\langle I(t)I(t+\tau )\rangle /{\left\langle I(\tau )\right\rangle }^{2}$, of a single speckle. The decay of the autocorrelation curve is fit with the solution of the correlation diffusion equation^4^ to obtain an index of blood flow (${\mathrm{BF}}_{i}$) in units of ${\mathrm{cm}}^{2}/s$. Although the units of ${\mathrm{BF}}_{i}$ are not the conventional units of ml/min/100  g tissue for perfusion, ${\mathrm{BF}}_{i}$ has been shown to be reliably proportional to absolute flow, as demonstrated against “gold standards,” such as arterial spin-labeled MRI,^8^^–^^10^ fluorescent microspheres,^11^ bolus tracking time-domain NIRS,^12^^,^^13^ and phase-encoded velocity mapping MRI.^14^

A significant limitation of both NIRS and DCS is the limited depth sensitivity, which is crucial when aiming to measure cerebral hemodynamics noninvasively in humans. NIRS cerebral oximetry measurements of hemoglobin oxygenation (${\mathrm{SO}}_{2}$) are contaminated by scalp and skull physiology, and the resulting ${\mathrm{SO}}_{2}$ includes extracranial contributions.^15^^–^^18^ This uncertainty on the cerebral origin of the signal limits the widespread adoption of NIRS cerebral oximeters in the clinical practice.^19^^,^^20^ Similarly, in functional NIRS imaging studies, the low-depth sensitivity not only limits the method of studying the superficial cortical regions but also reduces the contrast of the measured evoked hemodynamic changes. DCS is more sensitive to faster flow, and blood flow in the brain is usually 4 to 6 times higher than scalp blood flow,^21^ which should make DCS more sensitive to brain hemodynamics than NIRS.^22^ However, this would hold true only if the signal-to-noise ratio (SNR) of the two modalities were comparable.

In this paper, we demonstrate that SNR strongly impacts effective DCS depth sensitivity and, through the use of longer wavelengths than what has conventionally been used in NIRS, show how we can substantially improve DCS SNR and hence sensitivity to deep tissue blood flow. The use of longer wavelengths to increase light penetration^23^ has already been applied in the microscopic imaging domain by modalities such as multiphoton microscopy,^24^^,^^25^ optical coherence tomography,^26^ and photoacoustic imaging.^27^ Here we show theoretically and experimentally the advantages of operating DCS at 1064 nm, we highlight current hardware component limitations, and we report initial feasibility of DCS measurements in humans at this wavelength, comparing performances with DCS in the typical NIRS wavelength range. In particular, we demonstrate for the first time the ability to make high-speed cardiac pulsation-resolved measurements with high-deep-tissue sensitivity, achieved through large (3 cm) source–detector separations for continuous wave DCS and late temporal gates (1 ns start time) for time-domain DCS.

## 2 Theoretical Advantages of DCS at 1064 nm versus 765 to 850 nm

### 2.1 Light Attenuation Coefficient and Depth Penetration

**Fig. 1:**
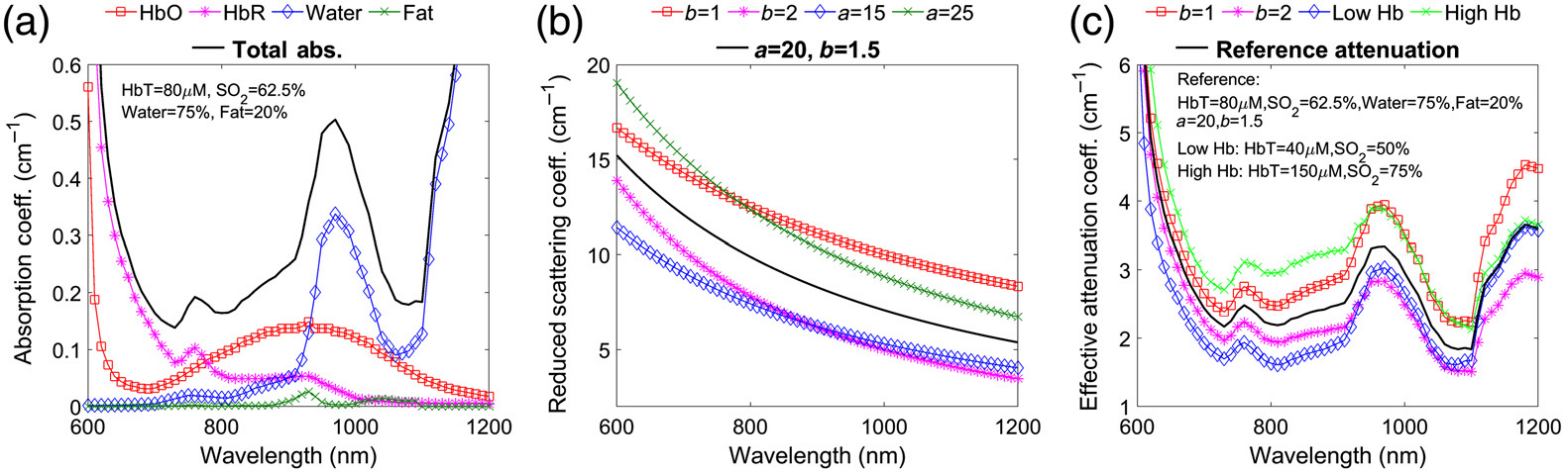
(a) Absorption spectra of main chromophores in brain tissue between 600 and 1200 nm assuming HbT=80  μM, ${\mathrm{SO}}_{2}=62.5\%$, 20% volume fraction of fat and 75% volume fraction of water: oxy-hemoglobin (HbO), red squares; deoxy-hemoglobin (HbR), magenta stars; water, blue diamonds; and fat, green crosses. The thick black line is the resulting total absorption. Absorption of melanin, cytochrome oxidase, and other possible chromophores is below $10^{-3}\;{\mathrm{cm}}^{-1}$ in most biological tissues. For shorter wavelengths, hemoglobin absorption increases; for longer wavelengths, water absorption further increases. (b) Reduced scattering coefficient assuming a power law wavelength dependence with a=20 and b=1.5 for $\lambda_{0}=500\;\mathrm{nm}$, thick black line, and changing a and b within physiological ranges, colored lines with symbols. (c) Resulting effective attenuation coefficient: thick black line attenuation is derived from the total absorption of (a) and reduced scattering coefficient with a=20 and b=1.5 for $\lambda_{0}=500\;\mathrm{nm}$; for red squares and magenta stars, we assumed b=1 and = 2, respectively; blue diamonds for low hemoglobin, assuming HbT=40  μM and ${\mathrm{SO}}_{2}=50\%$; green crosses for high hemoglobin, assuming HbT=150  μM and ${\mathrm{SO}}_{2}=75\%$.

Absorption in biological tissues is dominated by hemoglobin in the ultraviolet and visible spectral regions (200 to 650 nm) and by water for wavelengths above 900 nm.^28^ Between 650 and 900 nm, absorption is more than one order of magnitude lower, and NIRS uses this transmission window to investigate tissue several cm below the surface. Between 1050 and 1100 nm, the water spectrum has a local minimum [Fig. 1(a)], offering an additional transmission window for deep tissue measurements. Above 1100 nm, water absorption continues to increase with peaks and minima well above the 1050 to 1100 nm values. Figure 1(a) shows the absorption spectra of the main tissue chromophores between 600 and 1200 nm using assumptions typical for the brain. For the hemoglobin absorption, we assumed a concentration (HbT) of 80  μM, an oxygenation (${\mathrm{SO}}_{2}$) of 62.5%, and used extinction coefficients compiled by Prahl et al.^29^ as well as by Bosschaart et al.^30^ For the water absorption, we used values from Hale et al.^31^ and assumed a 75% volume fraction in tissue.^32^ For fat absorption, we used values from van Veen et al.^33^ and assumed a 20% volume fraction.^34^ Under these assumptions, the total absorption coefficients ($\mu_{a}$) at 765, 785, and 850 nm, wavelengths commonly used for DCS, are 0.19, 0.17, and $0.20\;{\mathrm{cm}}^{-1}$, respectively, very similar to the total absorption at 1064 nm ($0.18\;{\mathrm{cm}}^{-1}$). By varying the hemoglobin concentration between 40 and 120  μM, hemoglobin oxygenation between 40% and 95%, fat content between 0% and 40%, and water content between 55% and 95%, on average, we obtain absorption coefficients equal to the one in our example (see Table 1).

**Table 1:** Mean and standard deviation of optical properties across the range of chromophore concentrations considered in simulation.

λ (nm)	$\mu_{a}$ (${\mathrm{cm}}^{-1}$)	$\mu_{s}^{\prime }$ (${\mathrm{cm}}^{-1}$)	$\mu_{\mathrm{eff}}$ (${\mathrm{cm}}^{-1}$)
765	0.19±0.08	9.2±3.3	2.2±0.8
785	0.17±0.07	8.8±3.2	2.1±0.7
850	0.20±0.08	8.0±3.0	2.1±0.7
1064	0.18±0.04	6.0±2.4	1.7±0.5

In biological tissues, the reduced scattering coefficient ($\mu_{s}^{\prime }$) decreases with wavelength and follows the empirical power law relationship of $\mu_{s}^{\prime }(\lambda )=a{\left(\lambda /\lambda_{0}\right)}^{-b}$, where a is a scaling factor at wavelength $\lambda_{0}$ and b is the scattering power. The scattering spectra for typical values of a=20 at $\lambda_{0}=500\;\mathrm{nm}$ and b=1.5 (black line)^28^^,^^33^ are shown in Fig. 1(b), together with additional example spectra assuming a=15, a=25, b=1, or b=2. Similarly, we varied the scattering parameters over a wide range of 8<a<25 and 0.5<b<2.4 and calculated that on average at 1064 nm $\mu_{s}^{\prime }$ is 35%, 32%, and 25% lower than at 765, 785, and 850 nm, respectively (Table 1).

The cumulative effect of absorption and scattering on light propagation in the diffusive regime is given by the effective attenuation coefficient [$\mu_{\mathrm{eff}}(\lambda )=\sqrt{3\mu_{a}(\mu_{a}+\mu_{s}^{\prime })}$]. Figure 1(c) reports $\mu_{\mathrm{eff}}$ spectra for typical absorption and scattering [black lines in (a)–(c)], for high- and low-scattering power (b=1 red squares and b=2 magenta stars), and for low and high hemoglobin concentration (40  μM: blue diamonds and 150  μM: green crosses). Over the wide ranges of absorption and scattering described above, the average effective attenuation coefficient for diffuse light at 1064 nm is 23%, 21%, and 21% lower than at 765, 785, and 850 nm, respectively (Table 1).

In the examples of Fig. 1, we used a high content of water (75%), typical of brain tissue.^32^ For biological tissues with lower water content, the reduction in the effective attenuation at 1064 nm is even larger, despite the increase in fat content, which has a negligible contribution.

**Fig. 2:**
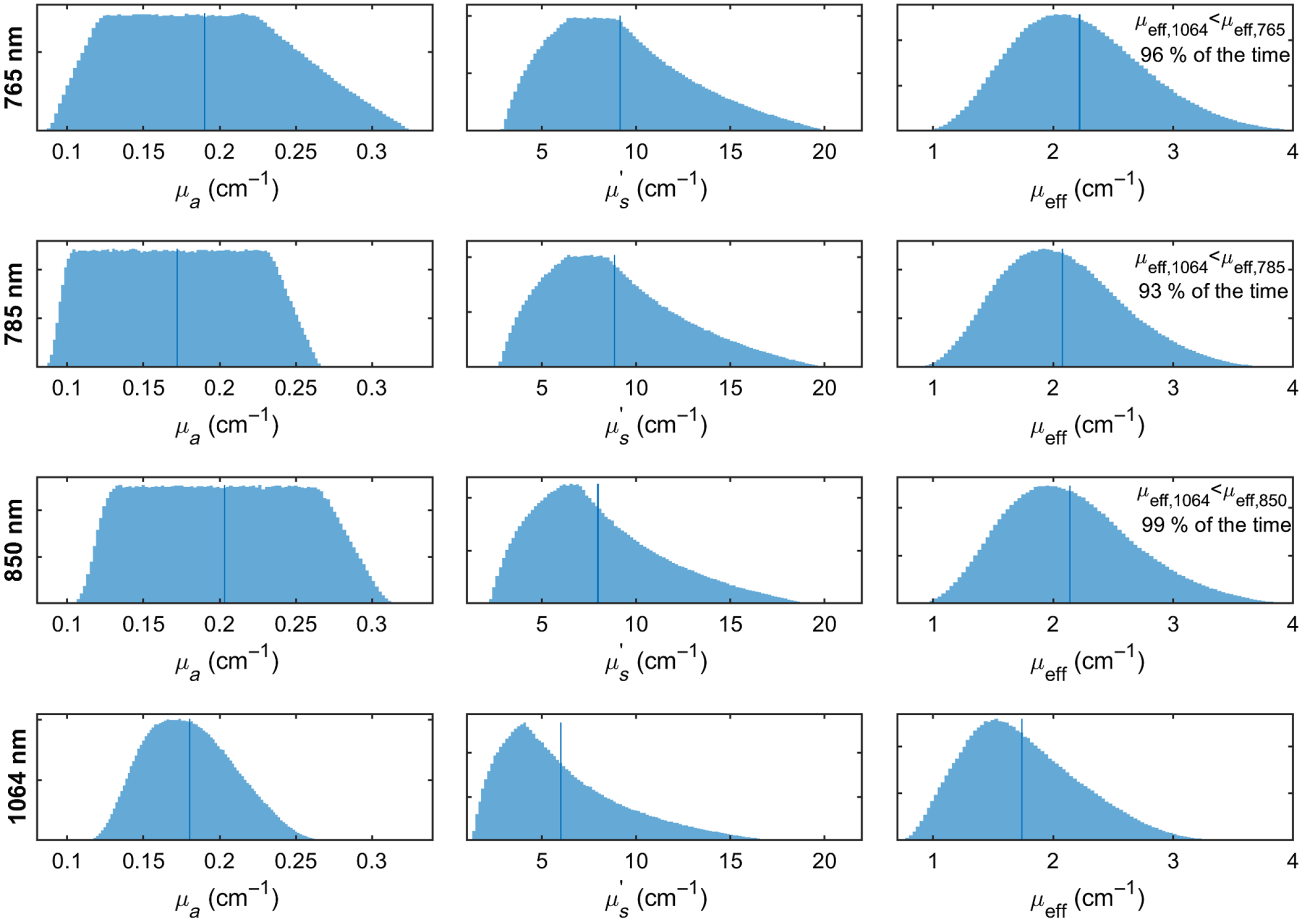
Histograms of absorption, reduced scattering, and effective attenuation coefficients at 765, 785, 850, and 1064 nm, respectively, randomly sampled (N=150,000) for 40  μM<HbT<120  μM, $40\%<{\mathrm{SO}}_{2}<85\%$, $0.55<f_{H2O}<0.95$, $0<f_{\text{fat}}<0.4$, 8<a<25, 0.5<b<2.4 to cover the vast majority of possible circumstances for tissue measurements. Vertical bars indicate average values. In pairwise comparisons (across the set of randomly sampled parameters), $\mu_{\mathrm{eff},1064}$ was lower than $\mu_{\mathrm{eff},765}$, $\mu_{\mathrm{eff},785}$, and $\mu_{\mathrm{eff},850}$ in 96%, 93%, and 99% of the time, respectively, indicating 1064 nm nearly always offers lower attenuation than the shorter wavelengths commonly used for DCS measurements.

Figure 2 presents histograms of the optical absorption, reduced scattering, and effective attenuation coefficients at 765, 785, 850, and 1064 nm, respectively, across 150,000 randomly sampled scenarios from the tissue chromophore concentration and scattering parameter ranges given above. Of note, when compared in a pairwise fashion across wavelengths for each randomly chosen chromophore concentration and scattering parameter choice set, the effective attenuation coefficient was lower at 1064 nm than at 765, 785, and 850 nm in 96%, 93%, and 99% of the time, respectively.

A lower effective attenuation coefficient implies a larger number of photons traveling through the tissue will be able to reach the detector. In fact, in the diffusive regime, the relationship between the detected intensity at two wavelengths is

$$I_{\lambda_{2}}/I_{\lambda_{1}}=e^{(\frac{\mu_{{\mathrm{eff}}_{\lambda_{1}}}-\mu_{{\mathrm{eff}}_{\lambda_{2}}}}{\mu_{{\mathrm{eff}}_{\lambda_{1}}}})\cdot r},$$

and for r=3  cm, $\mu_{\mathrm{eff}\;}$ $(765\;\mathrm{nm})=2.2\pm 0.8\;{\mathrm{cm}}^{-1}$, $\mu_{\mathrm{eff}}$ $(785\;\mathrm{nm})=2.1\pm 0.7\;{\mathrm{cm}}^{-1}$, $\mu_{\mathrm{eff}}$ $(850\;\mathrm{nm})=2.1\pm 0.7\;{\mathrm{cm}}^{-1}$, and $\mu_{\mathrm{eff}}$ $(1064\;\mathrm{nm})=1.7\pm 0.5\;{\mathrm{cm}}^{-1}$, the number of photons available at the detector at 1064 nm is, on average, 1.7 to 2.0 times larger than at wavelengths between 765 and 850 nm. Further, the lower attenuation results in longer (thus deeper) photon pathlengths being detectable, extending the penetration depth of the DCS measurement.

The fact that hemoglobin absorption is low at 1064 nm makes this wavelength choice impractical for NIRS, but this is not an issue for DCS as DCS contrast arises from the dynamic scattering of light by moving red blood cells, which is still high at 1064 nm. The lower scattering at 1064 nm does result in a somewhat reduced sensitivity to motion; however, this is more than compensated for by the other favorable aspects of using 1064 nm operation, as demonstrated below.

### 2.2 Overall Photon Budget Considerations

In addition to the lower attenuation, other factors contribute to the larger number of detectable photons at 1064 nm than at conventional DCS wavelengths.

DCS, like NIRS, needs to be performed in compliance with the ANSI standards limits for safe skin exposure (ANSI Z136.1) to be categorized as a nonsignificant risk device; thus, it needs to irradiate the tissue with low-power light. Based on these standards, the maximum permissible exposure (MPE) of skin by optical illumination are ${\mathrm{MPE}}_{765}=0.27\;W/{\mathrm{cm}}^{2}$, ${\mathrm{MPE}}_{785}=0.29\;W/{\mathrm{cm}}^{2}$, ${\mathrm{MPE}}_{850}=0.40\;W/{\mathrm{cm}}^{2}$, while from 1000 to 1400 nm ${\mathrm{MPE}}_{1000-1400}=1.07\;W/{\mathrm{cm}}^{2}$. Per the standard, for an illumination spot larger than 1-mm-diameter, a 3.5-mm aperture can be applied, translating to a light power of 26 mW at 765 nm, 28 mW at 785 nm, 38 mW at 850 nm, and 103 mW at 1064 nm. This means that at 1064 nm we can deliver 2.7 to 4 times more energy than at wavelengths between 765 and 850 nm. Moreover, because photons at longer wavelengths carry less energy $E_{\lambda_{2}}=E_{\lambda_{1}}\cdot \frac{\lambda_{1}}{\lambda_{2}}$, at longer wavelengths this increases the number of photons per unit of energy. Hence at 1064 nm, we can emit 1.3 to 1.4 times more photons per unit of illumination power than at wavelengths between 765 and 850 nm.

Multiplying together these three factors, i.e., the lower effective attenuation, the higher MPE, and the larger number of photons per unit of energy, using a 3-cm source–detector separation at 1064 nm, we end up with 13, 10.5, and 7 times more photons at the detector than at 765, 785, and 850 nm, respectively. This is a considerable gain as the number of photons directly impacts the measurement SNR.

### 2.3 Intensity Autocorrelation Noise Considerations

**Fig. 3:**
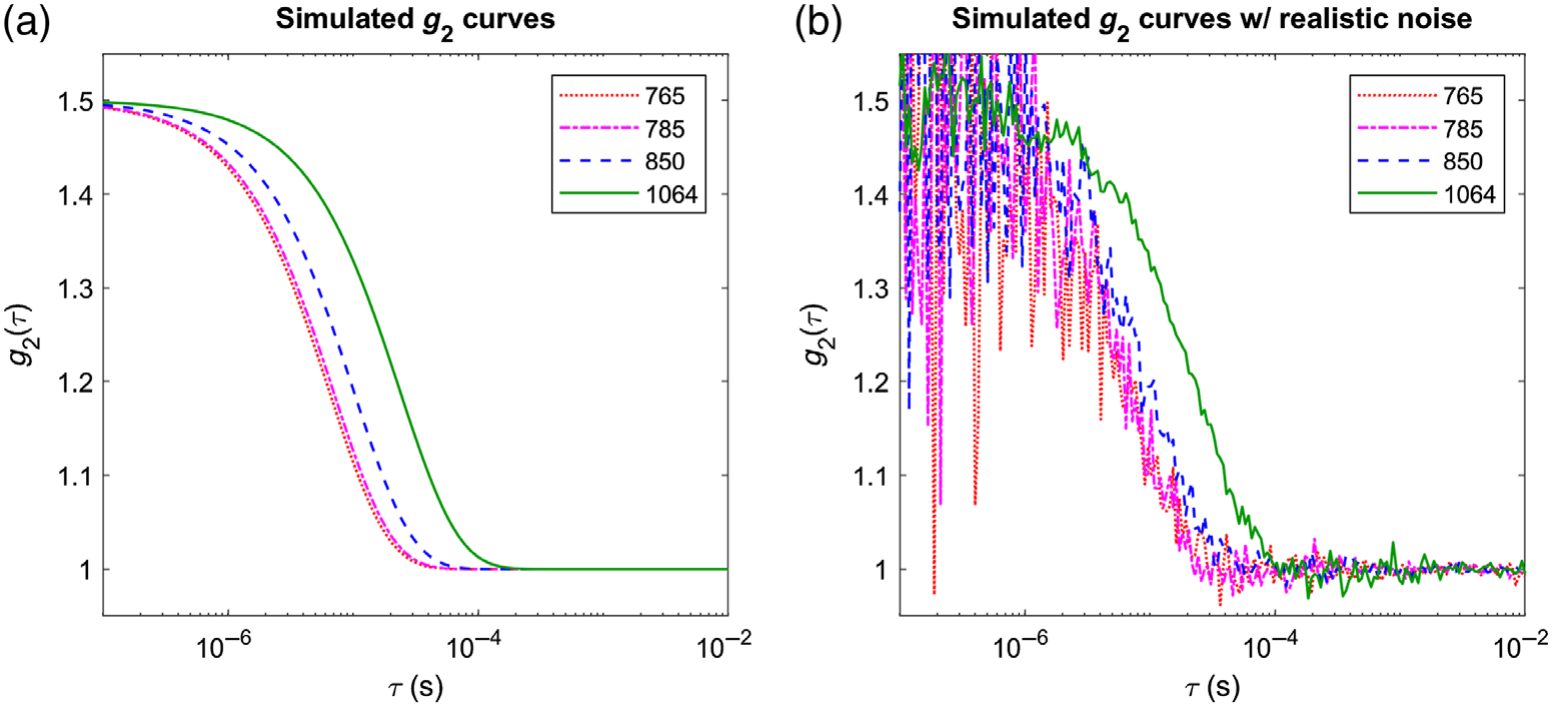
Simulated $g_{2}$ curves for measurements with 3-cm source–detector separation on a homogeneous sample with optical properties matching Table 1, assuming β=0.5, an integration time of 10 s, and a blood flow index ${\mathrm{BF}}_{i}=2\times 10^{-8}\;{\mathrm{cm}}^{2}/s$: (a) noise free; (b) with realistic noise added assuming a 7-kcps count rate at 765 nm, 8.05 kcps at 785 nm, 10.9 kcps at 850 nm, and 42.5 kcps 1064 nm on each of 4 co-located fibers.

Figure 3(a) shows example $g_{2}$ curves for a 3-cm source–detector separation, using the optical properties in Table 1 and assuming a blood flow index, ${\mathrm{BF}}_{i}=2\times 10^{-8}\;{\mathrm{cm}}^{2}/s$. The lower scattering and longer wavelength result in a later autocorrelation decay at 1064 nm versus shorter wavelengths, moving the correlation transition region to longer delay time bins where the statistical fluctuation noise is lower at a given photon count. Figure 3(b) shows updated $g_{2}$ simulations in which all factors discussed so far are taken into account to generate curves with realistic noise based on the DCS correlation noise model from Zhou.^35^ To this end actual photon counts needed to be specified, which in fact is one of the major advantages of operating at 1064 nm. Hence, we assumed a photon count rate of 7 kcps through a single-mode fiber for the 765-nm measurement, estimated from our experience with *in vivo* measurements. We simulated measuring with four co-located detector fibers to increase SNR^36^—as is often done in practice. This photon count rate was scaled up conservatively by just half of the factors estimated in Sec. 2.2 at the other wavelengths and considered 8050 counts per second at 785 nm, 10,900 counts per second at 850 nm, and 42,500 counts per second at 1064 nm. A 10-s averaging time was assumed. It is apparent from this simulation that, even under these conservative assumptions, 1064 nm measurements benefit from a substantial increase in SNR.

### 2.4 Monte Carlo Simulations of Sensitivity to Deep Blood Flow Change in the Presence of Realistic Noise

To further give a realistic assessment of the expected performance of DCS at 1064 nm in practice, we used Monte Carlo simulations in conjunction with a correlation noise model^35^ and typical experimental parameters to estimate the level of change in cerebral blood flow that can be detected with 95% confidence and 80% power. To this end, we considered a two-layered slab, with a superficial layer thickness of 1 cm and ran separate simulations corresponding to 765, 850, and 1064 nm illumination, respectively. For the two layers, we assumed typical hemoglobin and water concentrations as reported in the literature.^37^ Specifically, we assumed that the superficial layer contains 30  μM HbT, with a 66% ${\mathrm{SO}}_{2}$ and has a water fraction of 0.6. We assumed that the deep (cerebral) layer contains 80  μM HbT with a 62.5% ${\mathrm{SO}}_{2}$ and has a water fraction of 0.75 (as in Fig. 1). Optical absorption was derived from these chromophore concentrations using published extinction coefficients at each wavelength as detailed in the previous sections. Optical scattering was assumed to be homogeneous, equal to 9, 8, and $6\;{\mathrm{cm}}^{-1}$, respectively, at the three wavelengths, following the average optical scattering derived in the previous section (as shown in Table 1). A blood flow index of $1\times 10^{-8}\;{\mathrm{cm}}^{2}/s$ was assumed for the superficial layer, and a blood flow index of $6\times 10^{-8}\;{\mathrm{cm}}^{2}/s$ was assumed for the deep layer, similar to our previous publication.^22^ To assess sensitivity to changes in blood flow in the deeper layer, the deep flow was increased in turn by amounts from 1% to 100% in steps of 1%. We fixed the source–detector separation to 3 cm, the largest source–detector separation commonly used for *in vivo* measurements with existing DCS systems.

Electric field autocorrelation functions $g_{1}=\langle E(t)E^{*}(t+\tau )\rangle /\langle E(t)E^{*}(t)\rangle$ were derived from photon pathlengths and momentum transfer accumulations in each tissue type using the MCX simulation package.^38^ $g_{1}$ curves were then converted to intensity autocorrelation $g_{2}$ curves using the Siegert relationship $g_{2}=1+\beta$. $g_{1}^{2}$ with β=0.5 corresponding to polarization-insensitive detection.

The Monte Carlo generated $g_{2}$ curves were further modified to add realistic noise levels based on the model of Zhou.^35^ Hence, taking into account the photon budget considerations discussed in Sec. 2.2, we used the same fluence values as used in Sec. 2.3 and assumed four co-located fibers as well. The noise model predicted standard deviations for $g_{2}$ at each lag time were applied by randomly sampling a normal distribution. A total of 60 noise realizations were obtained at each flow level, and the integration time in the noise model was set to 1 s, i.e., we simulated 60 1-s measurements at each flow level.

To generate population-based statistics, each sequence of 60 “measurements” (at baseline or at elevated deep flow levels, respectively) was divided into six 10-s segments. Each baseline segment was combined with each elevated flow segment at each flow level providing a population of 36 “trials” at each deep flow change level. Thus each trial was an attempt to detect a statistically significant change (p<0.05) in deep flow by comparing 10 s of baseline data with 10 s of elevated flow data, both acquired with a 1-s integration time. We consider this to be a realistic scenario for neuromonitoring in clinical practice, for example.

**Fig. 4:**
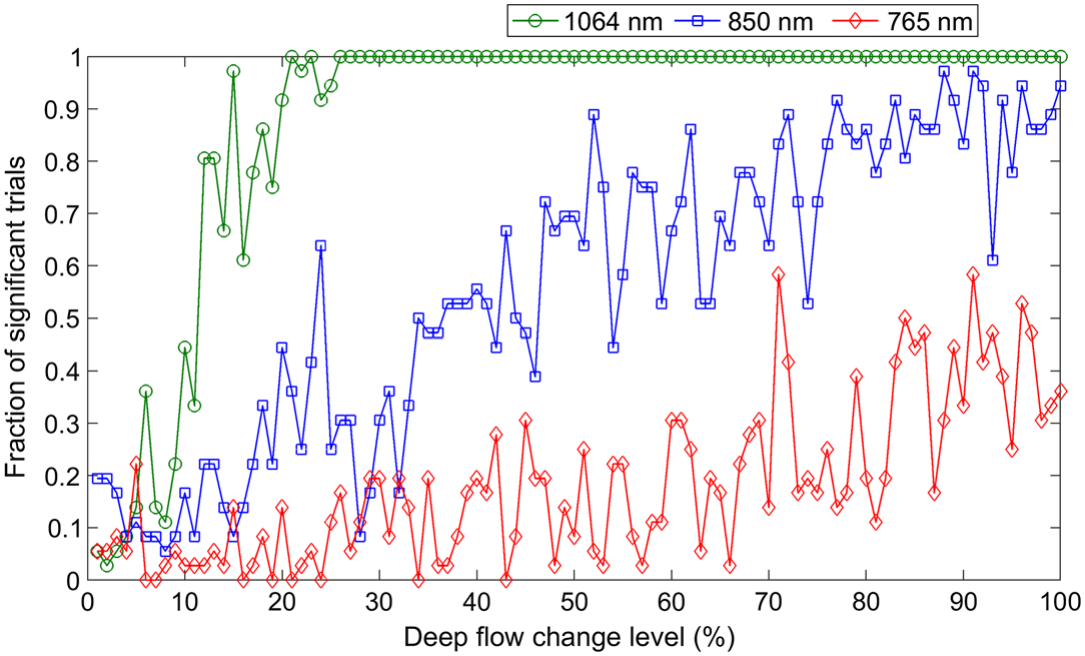
Probability of detecting a statistically significant change in deep-tissue blood flow from a pair of 10 s measurements as a function of the actual amount of change for noise-realistic simulated measurements at 765 nm (red diamonds), 850 nm (blue squares), and 1064 nm (green circles). We assumed 30  μM HbT, with a 66% ${\mathrm{SO}}_{2}$, water fraction of 0.6 (no fat), and ${\mathrm{BF}}_{i}=10^{-8}\;{\mathrm{cm}}^{2}/s$ for the superficial layer (1-cm thick), and 80  μM HbT with a 62.5% ${\mathrm{SO}}_{2}$, water fraction of 0.75 (also with no fat), and ${\mathrm{BF}}_{i}=6\times 10^{-8}\;{\mathrm{cm}}^{2}/s$ for the deep layer. The source–detector separation was set to 3 cm, and we assumed 7 kcps at 765 nm on four co-located fibers, scaling up to 10.9 and 42.5 kcps at 850 and 1064 nm, respectively, reflecting conservative estimates of the benefits of longer wavelengths.

Figure 4 shows the results of the simulations. DCS at 765 nm (red diamonds) is able to detect significant changes in blood flow in, at best, 30% to 40% of the trials even for the highest level of simulated deep blood flow changes (above 80% with respect to baseline). DCS at 850 nm (blue squares) performances are somewhat better, though deep flow changes above 75% are needed to achieve an 80% detection rate of deep flow change. By comparison, the performance of DCS at 1064 nm (green circles) is dramatically better with more than 80% of the trials able to detect a statistically significant change in deep blood flow of 20% or more.

## 3 Experimental Demonstration of DCS at 1064 nm and Comparison with DCS at 765 to 850 nm

### 3.1 DCS Hardware at 1064 nm

#### 3.1.1 CW-DCS system

For our measurements, we used a custom-built multicolor DCS system^39^ built in house and replaced one of the sources with a laser at 1064 nm. Specifically, we kept the monolithic distributed Bragg reflector (DBR) lasers at 765 and 850 nm (PH765DBR and PH850DBR by Photodigm Inc.) and replaced the laser at 808 nm with a DBR laser emitting at 1064 nm (PH1064DBR). The same custom circuitry was used to drive this laser, without fast multiplexing between the three lasers, allowing for operation at a wavelength manually selected each time. Later experiments used a 785-nm laser from CrystaLaser due to a hardware issue with the 765-nm Photodigm device. For the detectors, this system uses a single-photon avalanche diode (SPCM-850-14-FC, by Excelitas Technologies), which has a good photon detection efficiency (PDE) at 765 and 850 nm (64% and 54%, respectively) but only 3% efficiency at 1064 nm. The low PDE of the silicon detectors at 1064 was a limiting factor on the choice of source–detector separation and was taken into account when comparing performances at different wavelengths.

An alternative configuration used a TOPTICA eagleyard distributed feedback EYP-DFB-1064 laser diode as a source, amplified using a Cybel MantaRay fiber amplifier. The detectors in this case were either Photon Spot or Quantum Opus superconducting nanowire single-photon detectors (SNSPDs) with greater than 90% PDE at 1064 nm, the output from which was patched into our DCS system’s correlator board. The use of SNSPDs was necessitated by the shortcomings of commercially available InGaAs SPADs that have unacceptably long afterpulsing, which prevents the effective measurement of the autocorrelation decay at longer source–detector separations needed to be sensitive to brain blood flow.

#### 3.1.2 TD-DCS system

To acquire proof-of-concept data using time-domain diffuse correlation spectroscopy (TD-DCS) at 1064 nm, we modified our previously reported portable TD-DCS system^40^ to replace the source and detection components, while reusing the time-tagging and correlation hardware. We used a 1064-nm Picoquant seed laser emitting ∼650  ps FWHM pulses with a repetition rate of 80 MHz at an average power of about 5 mW. The output of the seed laser was coupled into a Cybel MantaRay fiber amplifier. The same Photon Spot or Quantum Opus SNSPD detector described above was used to capture the light returned from tissue.

### 3.2 Phantom Measurements

**Fig. 5:**
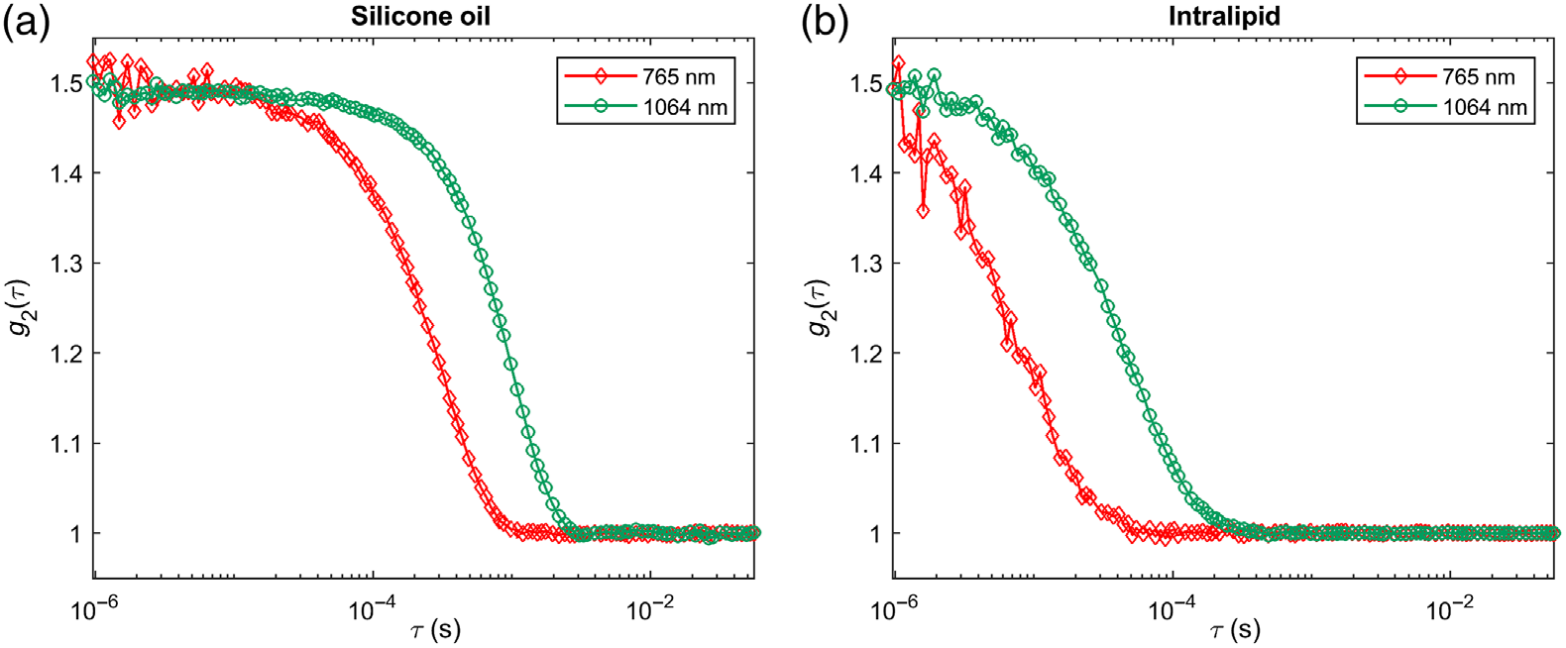
Intensity autocorrelation function ($g_{2}$) measurements at 765 nm (red diamonds) and 1064 nm (green circles) in (a) a silicone oil phantom and (b) an intralipid phantom.

Figure 5 shows the CW-DCS temporal autocorrelation functions for 3-cm source–detector separation measured at 765 nm (red diamonds) and 1064 nm (green circles) in (a) silicone oil and (b) intralipid. The decay of $g_{2}$ is much slower (occurs at later time lags) for the two homogeneous liquid phantoms at 1064 nm because of the lower scattering and higher wavelength. If we calculate ${\mathrm{BF}}_{i}$ assuming reduced scattering values of $8\;{\mathrm{cm}}^{-1}$ at 765 nm and $6\;{\mathrm{cm}}^{-1}$ at 1064 nm, we obtain the same ${\mathrm{BF}}_{i}$ in the two phantoms.

**Fig. 6:**
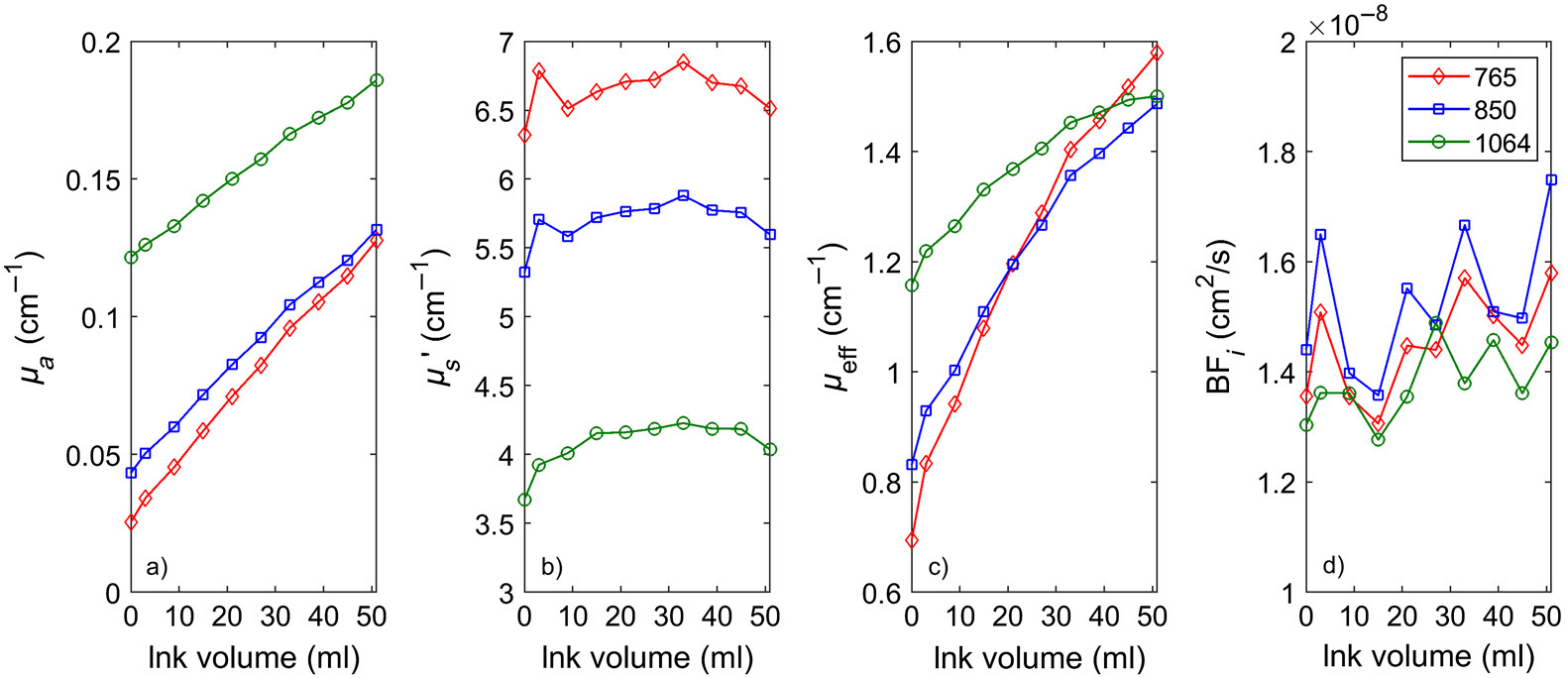
CW-DCS measurements of ${\mathrm{BF}}_{i}$ (in this case, the Brownian diffusion coefficient $D_{b}$) in a liquid phantom across wavelengths and phantom optical properties. (a) Optical absorption values, (b) corresponding optical scattering values, (c) corresponding attenuation coefficient values, and (d) recovered ${\mathrm{BF}}_{i}$ taking into account actual optical properties.

Another set of liquid phantom titrations was used to further verify that the same dynamic properties are recovered no matter what wavelength is used to perform the measurements. The phantoms were constructed by adding India ink to a diluted intralipid suspension in water. Optical properties at 765 and 850 nm were measured using a frequency-domain NIRS device (ISS MetaOx). Optical properties at 1064 nm were extrapolated from measurements in the 660- to 830-nm range. As shown in Fig. 6, using CW-DCS at the three wavelengths, we were able to obtain agreement of 10% or better in the measured Brownian diffusion coefficient values across wavelengths when taking into account the actual optical properties. Of note, due to water absorption, significant amounts of ink needed to be added to obtain comparable absorption at 765 and 850 nm compared with 1064 nm.

### 3.3 *In Vivo* Measurements

We conducted several proof-of-concept experiments to demonstrate the feasibility of *in vivo* DCS data acquisition at 1064 nm and to exemplify the advantages of operating at this wavelength. Subject consent was obtained in accordance with the policies and guidelines of the Massachusetts General Hospital/Partners Healthcare Institutional Review Board.

#### 3.3.1 Muscle blood flow during arm cuff occlusion

**Fig. 7:**
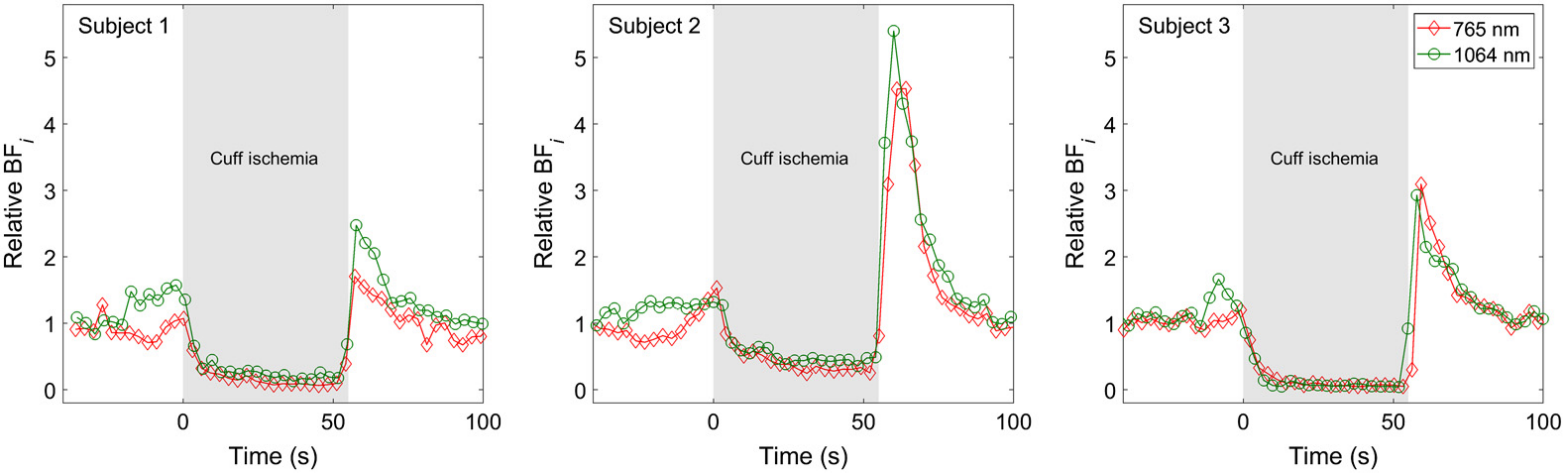
Measurements of muscle blood flow before, during, and after an arm cuff occlusion maneuver in three different subjects. Measurements at 765 nm (red diamonds) and 1064 nm (green circles) were conducted one after the other. Despite the nonsimultaneous nature of the measurement, there is very good agreement between measurements taken in the same subject.

As a first demonstration, we conducted an arm cuff occlusion measurement on three healthy volunteers using both 765 nm and 1064 nm CW-DCS systems. These tests were conducted using the Excelitas SPADs; thus, we chose a 1-cm source–detector separation to compensate for the low PDE at 1064 nm. Figure 7 shows the time courses of the relative changes in blood flow index measured at 765 and 1064 nm, normalized to the average of the first 20 s for the three different subjects. After 40 s of baseline, the blood pressure cuff was quickly inflated to 160 mmHg in each case. The pressure was then released about 55 s later. Two measurements were conducted in succession on each subject at 765 and 1064 nm, respectively. Scattering values of $8\;{\mathrm{cm}}^{-1}$ at 765 nm and $6\;{\mathrm{cm}}^{-1}$ at 1064 nm were chosen to obtain matching absolute blood flow index values at the baseline. Although the degree of blood flow reduction during occlusion and of reactive hyperemia when the pressure was released varied by subject, the DCS measurements at the two wavelengths agreed very well with each other, despite being conducted sequentially.

#### 3.3.2 Cerebral blood flow measurements

Measurements were obtained using both the CW-DCS and TD-DCS systems on the forehead of an adult volunteer. The 765-nm (or 785 nm) measurements used our standard systems,^39^^,^^40^ and we delivered 26 mW (28 mW at 785 nm) of average power on the skin surface. The 1064-nm measurements were conducted using the TOPTICA eagleyard single-frequency laser diode for CW-DCS and the Picoquant pulsed seed laser for TD-DCS, both amplified to 90 mW of average power using the Cybel MantaRay fiber amplifier. Either Photon Spot or Quantum Opus SNSPD detectors were used for both the CW-DCS and TD-DCS measurements at 1064 nm.

**Fig. 8:**
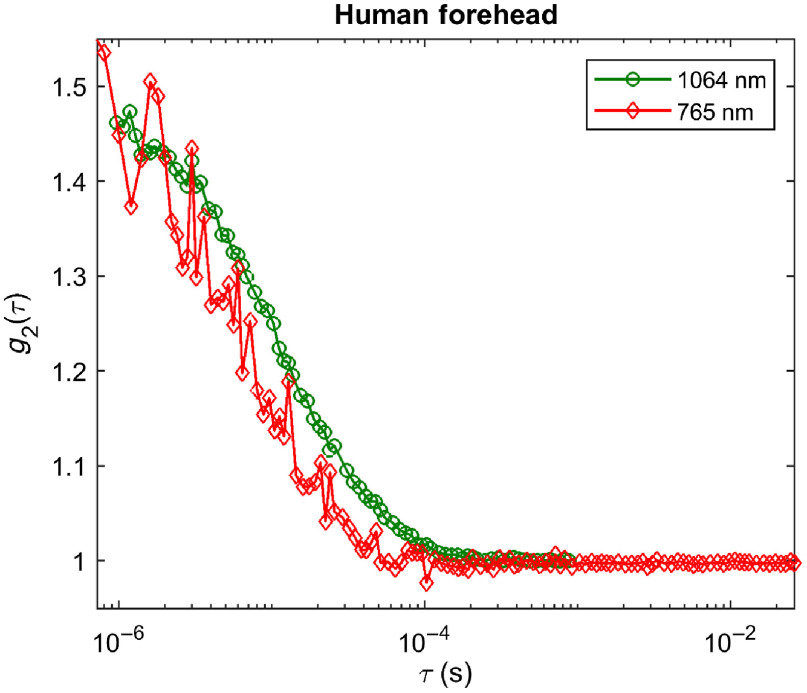
$g_{2}$ measurements at 765 nm (red diamonds) and 1064 nm (green circles) on the forehead of a human subject. The 1064-nm measurements show a later decay and significantly higher SNR.

Figure 8 shows the CW-DCS temporal autocorrelation functions for a 3-cm source–detector separation measured at 765 nm (red diamonds) and 1064 nm (green circles) on an adult human forehead. The difference between the $g_{2}s$ at the two wavelengths is smaller than in the phantom measurements shown in Fig. 5 (though remaining in favor of the 1064-nm measurement) because at 1064 nm light penetrates deeper and sees more of the brain blood flow. The higher sensitivity to the high perfusion in the brain “accelerates” the 1064-nm $g_{2}$, resulting in a more similar decay of $g_{2}$ at the two wavelengths. The 1064-nm measurement retains a significant SNR advantage due to the higher photon count (∼55  kcps at 1064 nm versus 7 kcps at 765 nm in this case, driven by higher photon availability and higher PDE of the SNSPD detector even with more brain tissue in the photon path). If we calculate ${\mathrm{BF}}_{i}$ assuming values of $8\;{\mathrm{cm}}^{-1}$ at 765 nm and $6\;{\mathrm{cm}}^{-1}$ at 1064 nm, we obtain a ${\mathrm{BF}}_{i}$ that is 50% higher at 1064 nm than at 765 nm in the forehead.

**Fig. 9:**
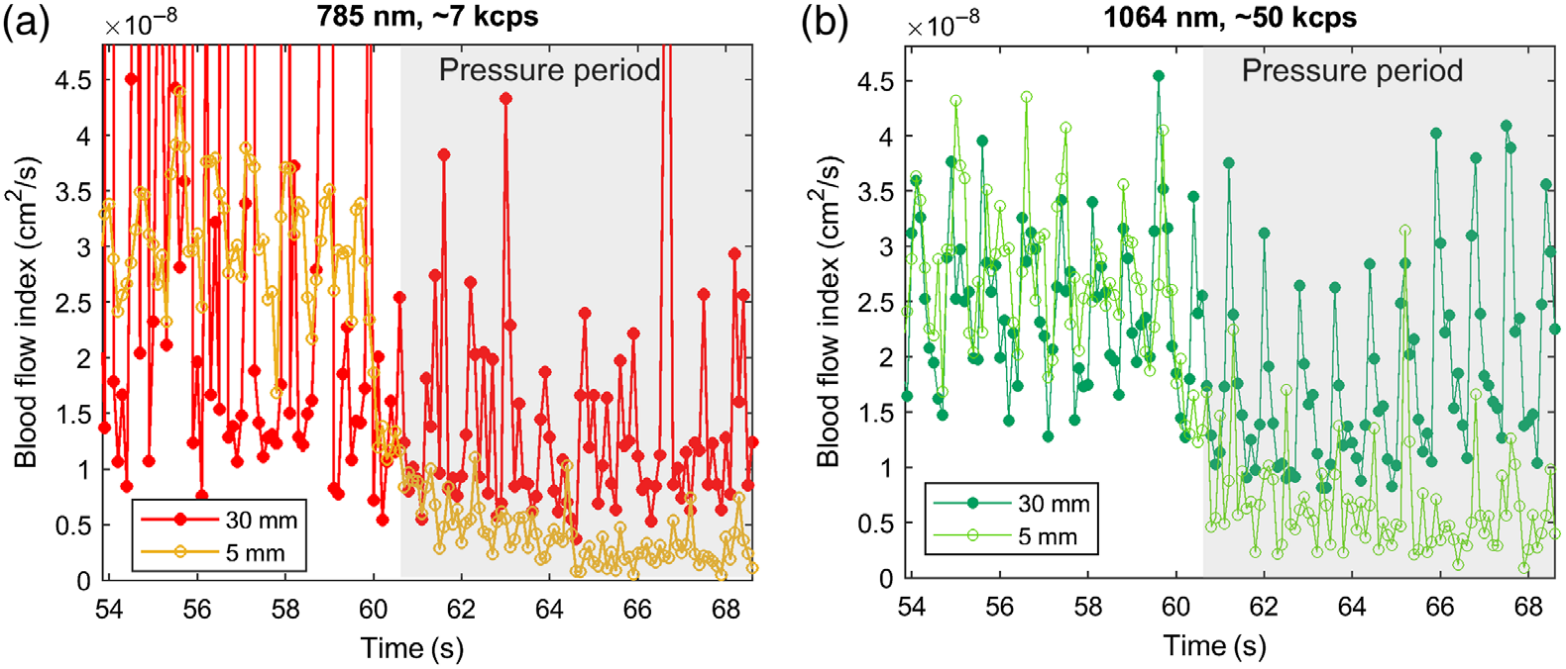
CW-DCS ${\mathrm{BF}}_{i}$ time course measurements during pressure modulation maneuvers on the forehead of a human subject using a 10 Hz acquisition rate at (a) 785 nm (orange/red symbols) and (b) 1064 nm (light/dark green symbols), respectively. The short separation (5 mm: orange/light green open circles, respectively) ${\mathrm{BF}}_{i}$ decreases as expected during the pressure period and is no longer pulsatile; cardiac pulsation remains apparent in the long separation channel (30 mm: red/dark green filled circles, respectively) at both wavelengths; however, only 1064 nm offers sufficient SNR to resolve the pulsation at a 3-cm source–detector separation.

Figure 9 shows the time course of CW-DCS ${\mathrm{BF}}_{i}$ at a 10-Hz acquisition rate, during a probe pressure modulation maneuver (marked by the gray shading starting just after the 60-s mark). By lightly pressing the optical probe onto the forehead, we reduce the blood flow to the skin under the probe. This results in a lower ${\mathrm{BF}}_{i}$ in the scalp and an unaffected ${\mathrm{BF}}_{i}$ in the brain. At a short source–detector separation (5 mm, orange/light green curves with open circles, respectively), we only measure ${\mathrm{BF}}_{i}$ in the scalp and the pressure modulation decreases ${\mathrm{BF}}_{i}$ substantially. At the same time, pulsatility is largely reduced. At a 3-cm separation (red/dark green curves with filled circles, respectively), we see a smaller reduction in average flow and pulsatility is preserved. Although evidence of cardiac cycle ${\mathrm{BF}}_{i}$ variation is visible at both wavelengths, the SNR of the 785-nm measurement is insufficient to resolve the pulsation, and the long-separation fit is rather unstable. At the same time, at 1064 nm we observe excellent SNR for the ${\mathrm{BF}}_{i}$ time course, easily resolving the pulsation, including finer features such as the dichroitic notch. These data demonstrate the ability to use 1064 nm DCS as a functional imaging technique with high temporal resolution.

**Fig. 10:**
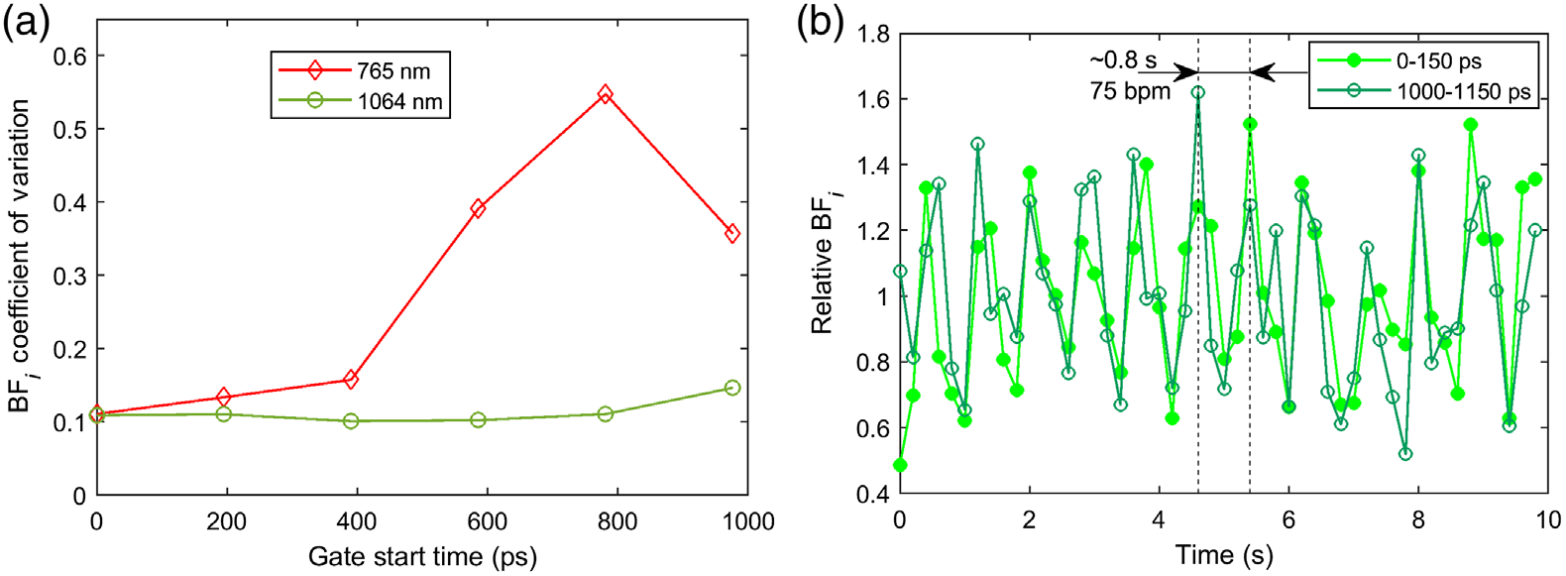
TD-DCS comparison between 765 and 1064 nm. (a) Comparison of the ${\mathrm{BF}}_{i}$ CoV versus gate start time for TD-DCS measurements conducted at 765 nm (red diamonds) and 1064 nm (green circles) using a 1-cm source–detector separation and the maximum power allowed by regulatory standards. (b) Evidence of cardiac pulsation in ${\mathrm{BF}}_{i}$ at a 5-Hz acquisition rate for early (0 to 150 ps, light green filled circles) and late (1000 to 1150 ps, dark green open circles) gates.

Figure 10 exemplifies TD-DCS measurements in the human forehead at 765 and 1064 nm using a 1-cm source–detector separation and employing the maximum power allowed by the regulatory standards. SNSPDs were used as the detector for both wavelengths. The data were processed at 5 Hz, after which the ${\mathrm{BF}}_{i}$ values were downsampled to 1/3  Hz to filter out cardiac pulsation and compute the coefficient of variation (CoV). The resulting ${\mathrm{BF}}_{i}$ CoVs versus gate start time for gates 150 ps wide are shown in Fig. 10(a) for 765 and 1064 nm. Although the CoV increases quicky versus gate at 765 nm and the fit becomes unstable by 600 ps after the peak of the temporal point spread function (TPSF), the 1064-nm curve remains relatively stable until 800 ps after the peak of the TPSF (likely dominated by physiological variability), and the CoV only shows a significant increase at 1 ns after the peak of the TPSF. Figure 10(b) shows the original 5 Hz data for 1064 nm TD-DCS measurements for an early gate (starting at the peak of the TPSF) and a late gate (starting 1 ns after the peak of the TPSF). The ${\mathrm{BF}}_{i}$ time traces clearly offer sufficient SNR to resolve the cardiac pulsation even at the late gate, exceeding any previously demonstrated TD-DCS measurements with sub 1 s integration times. The achieved SNR highlights the strong potential of TD-DCS as tool for functional brain imaging in addition to neuromonitoring applications.

## 4 Conclusion

DCS measurements at 1064 nm benefit from a multitude of advantages compared with DCS in the usual NIR range (765 to 850 nm) stemming from lower tissue attenuation, increased amount of light delivery allowed by regulatory standards, reduced energy per photon (enhancing available photon counts), and slower autocorrelation decay moving the transition regime to higher SNR delay time bins. We estimate these advantages through numerical simulations and demonstrate CW-DCS and TD-DCS measurements in phantoms and *in vivo*. These advantages are particularly important for adult brain neuromonitoring applications, for which DCS at 1064 nm can present a dramatic leap forward in performance and is likely to be much more robust, especially in older subjects (where the skin to brain distance may be larger due to brain tissue shrinkage^41^) and those with thicker extracerebral layers. A remaining challenge is the lack of suitable portable photon counting detectors. Potential solutions include developing custom detector designs, using cross-correlation configurations to remove InGaAs SPAD afterpulsing, or employing analog detection in conjunction with a heterodyne DCS approach as done at 850 nm by Zhou et al.^42^

## Code, Data, and Materials Availability

Data supporting the results reported in the manuscript can be requested by contacting the corresponding author.
